# Association between nutritional status scores and the 30-day mortality in patients with acute kidney injury: an analysis of MIMIC-III database

**DOI:** 10.1186/s12882-023-03329-5

**Published:** 2023-10-06

**Authors:** Tingting Gao, Xueyuan Yu

**Affiliations:** 1 Department of Comprehensive Medical, Shanxi Bethune Hospital, Shanxi Academy of Medical Sciences, Tongji Shanxi Hospital, Third Hospital of Shanxi Medical University, Taiyuan, 030032 Shanxi P.R. China; 2 Department of Nephrology, Qi Lu Hospital of Shandong University, No.107 Wenhua west road, Lixia District, Jinan, 250012 Shandong P.R. China

**Keywords:** AKI, PNI, GNRI, SOFA, SAPS-II, 30-day mortality, MIMIC-III

## Abstract

**Background:**

Studies have proven that the risk of acute kidney injury (AKI) increased in patients with malnutrition. Prognostic nutritional index (PNI) and geriatric nutritional risk index (GNRI) were general tools to predict the risk of mortality, but the prognostic value of them for in-hospital mortality among patients with AKI have not been validated yet. Herein, this study aims to explore the association between PNI and GNRI and 30-day mortality in patients with AKI.

**Methods:**

Demographic and clinical data of 863 adult patients with AKI were extracted from the Medical Information Mart for Intensive Care III (MIMIC-III) database in 2001–2012 in this retrospective cohort study. Univariate and multivariate Cox proportional regression analyses were used to explore the association between PNI and GNRI and 30-day mortality. The evaluation indexes were hazard ratios (HRs) and 95% confidence intervals (CIs). Subgroup analyses of age, Sequential Organ Failure Assessment (SOFA) score and Simplified Acute Physiology (SAPS-II) score were also performed.

**Results:**

Totally, 222 (26.71%) patients died within 30 days. After adjusting for covariates, PNI ≥ 28.5 [HR = 0.71, 95%CI: (0.51–0.98)] and GNRI ≥ 83.25 [HR = 0.63, 95%CI: (0.47–0.86)] were both associated with low risk of 30-day mortality. These relationships were also found in patients who aged ≥ 65 years old. Differently, high PNI level was associated with low risk of 30-day mortality among patients with SOFA score < 6 or SAPS-II score < 43, while high GNRI was associated with low risk of 30-day mortality among those who with SOFA score ≥ 6 or SAPS-II score ≥ 43 (all *P* < 0.05).

**Conclusion:**

PNI and GNRI may be potential predictors of 30-day mortality in patients with AKI. Whether the PNI is more recommended for patients with mild AKI, while GNRI for those with severe AKI is needed further exploration.

**Supplementary Information:**

The online version contains supplementary material available at 10.1186/s12882-023-03329-5.

## Background

Acute kidney injury (AKI) is defined by a sudden dysfunction of the kidney, and the in-hospital mortality of AKI is as high as 50% overall [1, 2]. AKI is related to adverse metabolic and nutritional outcomes such as metabolic abnormalities of protein and fat, inducing proinflammatory state and immune ability impairment [3]. The possible effects of nutritional conditions, substrate balance, and treatment processes cannot be neglected in hospitalization of patients with AKI [4].

The risk of AKI increased in patients with malnutrition [5]. Studies have showed that malnutrition in old patients with AKI was associated with high risk of in-hospital mortality [6, 7]. Therefore, assessment of nutritional status is essential for the identification of patients with AKI who have high risk of mortality. Among various nutritional assessment tools, prognostic nutritional index (PNI) and geriatric nutritional risk index (GNRI) have been reported to be associated with contrast-induced acute AKI [6, 8]. PNI is often used to evaluate postoperative outcomes and nutritional status in patients with malignancies [9], heart [10], kidney [11], and pulmonary [12] diseases. At the same time, GNRI is a general tool for predicting the risk of morbidity and mortality in elderly cancer patients [13]. Previous studies indicated that lower level of GNRI was associated with longer hospital stays, complications, and long-term mortality [14, 15]. However, the predictive value of PNI and GNRI of in-hospital mortality in patient with AKI have not been validated yet.

Herein, this study aims to explore the association between the PNI and GNRI and 30-day mortality in patients with AKI, and assess the predictive performance of them. We hope this study could provide some references for choosing optimal criteria for evaluation of the AKI prognosis, and to assist the clinical monitoring and treatment of AKI.

## Methods

### Study design and participants

Data of participants in this retrospective cohort study were extracted from the Medical Information Mart for Intensive Care III (MIMIC-III) database. The MIMIC-III was published by the computational physiology laboratory of Massachu-setts Institute of Technology (MIT, Cambridge, MA, USA), Beth Israel Deaconess Medical Center (BIDMC, Boston, MA, USA), and Philips Medical jointly. The clinical diagnosis and treatment information on more than 40,000 real patients who are predominantly White people living in the intensive care unit (ICU) of the BIDMC were collected and sorted out by MIMIC database since 2001. The publicly data are available on the website: https://mimic.mit.edu/.

A total of 863 adult patients with AKI, and hospitalized in the ICU at first admission for more than 1 day were initially included. Patients without the information of PNI, GNRI, oxygen saturation (SpO_2_), neutrophil lymphocyte ratio (NLR), or prothrombin time (PT) were excluded. Finally, 831 of them were eligible. Due to the MIMIC-III database was publicly available, and the written informed consent from participants has been obtained before the survey, no ethical approval of the Institutional Review Board (IRB) of Qi Lu Hospital of Shandong University was needed. Besides, all the study methods were performed in accordance with the relevant guidelines and regulations.

### Diagnosis of AKI

AKI diagnosis was according to the Kidney Disease Improving Global Outcomes (KDIGO) guidelines [16]: serum creatinine (Scr) increased by ≥ 0.3 mg/dL within 48 hours, or increased to ≥ 1.5 fold from baseline within the prior 7 days, or urine volume < 0.5 mL/kg/h for 6 hours or more.

### Definitions of PNI and GNRI

PNI was calculated by the method postulated by Onodera et al. [17]: [10 × albumin (gr/dL)] + [0.005× absolute preoperative lymphocyte count (per mm^3^)]. We obtained the optimum cut-off PNI value of 28.5 using the X-tile software [18], and then divided the PNI into two levels: PNI < 28.5 and PNI ≥ 28.5.

GNRI is an objective screening tool developed by Bouillanne et al. [19] to predict the nutrition-related complications in older persons. GNRI was calculated by the following formula: GNRI = [1.489× serum albumin (g/L)] + [41.7× (current weight in kilograms/ideal weight)]. Ideal weight was calculated using the Lorentz formulas [19]: height (cm)-100- ([height (cm) -150] /4) for men and height (cm)-100- ([height (cm)-150] /2.5) for women. When current weight exceeded ideal body weight, we set current weight in kilograms/ideal weight = 1. In addition, we classified GNRI into four levels according to the nutritional conditions: absent malnutrition (≥ 100), mild (97.50-99.99), moderate (83.50-97.49) and severe (< 83.50) malnutrition [20].

### Variables collection

The study variables were collected within the first 24 hours after ICU admission, including age, gender, race, mechanical ventilation use, vasopressors use, renal replacement therapy, AKI stage, ICU length of stay, Charlson comorbidity index (CCI), Sequential Organ Failure Assessment (SOFA) score, Simplified Acute Physiology Score II (SAPS-II), Glasgow Coma Scale (GCS), hemoglobin (HB), red blood cell distribution width (RDW), anion gap (AG), esti mated glomerularfiltrationrate (eGFR), SpO_2_, NLR, PT, lymphocytes count, neutrophil count, platelet count, blood glucose, international normalized ratio (INR), respiratory rate (RR), height, weight, and body mass index (BMI).

The BMI was calculated as the weight in kilograms divided by the square of the height in meters and was categorized into normal (18.5–25.0 kg/m^2^), overweight (25.0-29.9 kg/m^2^), obesity (≥ 30 kg/m^2^), and underweight (< 18.5 kg/m^2^). The eGFR was calculated according to CKD-EPI (mL/min/1.73m^2^) equation. NLR (neutrophil-lymphocyte ratio) = neutrophil count/ lymphocytes count.

### Outcome and follow-up

The study outcome was 30-day mortality. The MIMIC-III followed up patients’ survival status by information in the electronic medical charts and hospital department records, or making contact with the patients, their family members, their attending health care workers, or family physicians on the phone. The follow-up ended until patients died or 30 days after the ICU admission.

### Statistical analysis

We used the Kolmogorov-Smirnov test to test the normality of quantitative data. Normal distributed data were described by mean ± standard error (mean ± SE), and independent-samples t test for comparation between groups. Skewed distribution data were described by median and quartiles [M (Q1, Q3)], and Mann-Whitney U rank sum test was used for comparation. Frequency and composition ratio [N (%)] was used to describe the distribution of categorical data, and chi-square test (χ^2^) was used for cooperation.

We used the Spearman rank correlation analyses to assess the associations between PNI, GNRI, and eGFR, AKI stage, SOFA score, and SAPS-II score respectively. Univariate Cox regression model was used to screen the covariates. Univariate and multivariate Cox regression models were established to explore the relationships between PNI and GNRI and 30-day mortality in patients with AKI. Model 1 was the crude model. Model 2 adjusted for the sociodemographic variables including age, gender, and race. Model 3 additionally adjusted for mechanical ventilation use, vasopressors use, AKI stage, ICU length of stay, CCI, SOFA, SAPS-II, HB, RDW, AG, eGFR, SpO_2_, NLR, PT, GCS, INR, and RR basing on the Model 2. We also explored these associations in age (< 65 years old and ≥ 65 years old), SOFA (< 6 and ≥ 6) and SAPS-II (< 43 and ≥ 43) subgroups. The predictive performances of PNI and GNRI on 30-day mortality patients with AKI was assessed by Kaplan-Meierwith (KM) curve and C index.

The evaluation indexes were *r*_*s*_, hazard ratios (HRs) and 95% confidence intervals (CIs). Two-sided *P* < 0.05 is considered significant. Missing variables were deleted and the sensitivity analysis of characteristics of participants before and after deletion of missing data was showed in Table S1. Statistical analyses were by SAS 9.4 (SAS Institute., Cary, NC, USA).

## Results

### Characteristics of participants

**Fig. 1:**
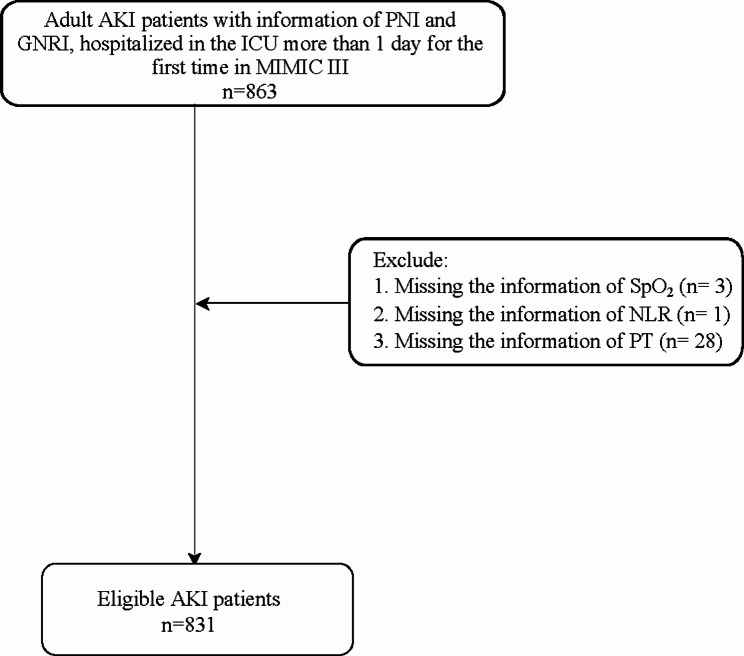
Flow chart of the participants screening

Figure 1 was the flowchart of participants screening. A total of 863 adult AKI patients with information of PNI and GNRI, and were hospitalized in the ICU over 1 day at the first admission were initially included. Then we excluded patients without information of SpO_2_ (n = 3), NLR (n = 1) or PT (n = 28). Finally, 831 of them were eligible.

Table 1 was the characteristics of AKI patients. Among the participants, 222 (26.71%) died within 30 days. The average age of patients was 64.61 years old, and 431 (51.87%) of them aged ≥ 65 years old. The median length of ICU stay was 4.25 days. Most of the patients had a PIN ≥ 28.5 (76.90%) and a GNRI ≥ 83.25 (57.52%). In addition, between survival group and 30-day mortality group, age, race, mechanical ventilation use, vasopressors use, AKI stage, CCI, SOFA, SAPS-II, HB, RDW, AG, eGFR, SpO_2_, NLR, PT, GCS, INR, RR, lymphocytes, follow-up time, PNI and GNRI of the patients were significantly different (all *P* < 0.05).

**Table 1:** Characteristics of AKI patients

Variables	Total (n = 831)	Survival (n = 609)	Mortality (n = 222)	Statistics	*P*
Age, years, Mean ± SD	64.61 ± 15.99	63.55 ± 16.18	67.52 ± 15.10	t=-3.19	0.001
Age groups, n (%)				χ^2^ = 5.44	0.020
Age < 65	400 (48.13)	308 (50.57)	92 (41.44)		
Age ≥ 65	431 (51.87)	301 (49.43)	130 (58.56)		
Gender, n (%)				χ^2^ = 2.51	0.113
Female	348 (41.88)	265 (43.51)	83 (37.39)		
Male	483 (58.12)	344 (56.49)	139 (62.61)		
Race, n (%)				χ^2^ = 17.06	0.002
Asian	12 (1.44)	10 (1.64)	2 (0.90)		
White	576 (69.31)	437 (71.76)	139 (62.61)		
Black	54 (6.50)	43 (7.06)	11 (4.95)		
Hispanic	21 (2.53)	17 (2.79)	4 (1.80)		
Others	168 (20.22)	102 (16.75)	66 (29.73)		
Mechanical ventilation use, n (%)				χ^2^ = 10.26	0.001
No	270 (32.49)	217 (35.63)	53 (23.87)		
Yes	561 (67.51)	392 (64.37)	169 (76.13)		
Vasopressors use, n (%)				χ^2^ = 41.60	< 0.001
No	416 (50.06)	346 (56.81)	70 (31.53)		
Yes	415 (49.94)	263 (43.19)	152 (68.47)		
Renal replacement therapy, n (%)				χ^2^ = 2.55	0.110
No	807 (97.11)	588 (96.55)	219 (98.65)		
Yes	24 (2.89)	21 (3.45)	3 (1.35)		
AKI stage, n (%)				χ^2^ = 84.21	< 0.001
I	145 (17.45)	126 (20.69)	19 (8.56)		
II	370 (44.52)	308 (50.57)	62 (27.93)		
III	316 (38.03)	175 (28.74)	141 (63.51)		
ICU length of stay, day, M (Q_1_, Q_3_)	4.25 (2.50,8.78)	4.26 (2.58,9.28)	4.16 (2.23,7.93)	Z=-1.28	0.202
CCI, M (Q_1_, Q_3_)	2.00 (1.00,4.00)	2.00 (1.00,4.00)	3.00 (2.00,5.00)	Z = 5.69	< 0.001
SOFA, M (Q_1_, Q_3_)	6.00 (4.00,9.00)	6.00 (3.00,8.00)	8.00 (5.00,11.00)	Z = 8.03	< 0.001
SAPS-II, M (Q_1_, Q_3_)	43.00 (33.00,55.00)	39.00 (30.00,50.00)	53.00 (43.00,66.00)	Z = 10.63	< 0.001
GCS, Mean ± SD	13.57 ± 2.95	13.85 ± 2.54	12.82 ± 3.76	t = 3.80	< 0.001
HB, g/dL, Mean ± SD	10.95 ± 2.20	11.11 ± 2.24	10.52 ± 2.03	t = 3.41	< 0.001
RDW, %, Mean ± SD	15.46 ± 2.32	15.18 ± 2.09	16.24 ± 2.71	t=-5.31	< 0.001
AG, mEq/L, Mean ± SD	15.60 ± 4.75	15.16 ± 4.34	16.82 ± 5.54	t=-4.03	< 0.001
eGFR, mL/min/1.73m^2^, M (Q_1_, Q_3_)	66.81 (37.33,96.98)	73.41 (42.16,100.45)	51.00 (31.21,86.23)	Z=-4.84	< 0.001
SpO_2_, %, Mean ± SD	96.65 ± 4.36	96.87 ± 4.13	96.05 ± 4.90	t = 2.21	0.028
NLR, M (Q_1_, Q_3_)	9.36 (5.80,17.40)	8.73 (5.25,15.30)	11.03 (7.18,21.95)	Z = 3.97	< 0.001
PT, seconds, M (Q_1_, Q_3_)	14.90 (13.50,17.70)	14.60 (13.40,16.90)	16.15 (14.10,20.70)	Z = 5.46	< 0.001
INR, M (Q_1_, Q_3_)	1.30 (1.20,1.70)	1.30 (1.20,1.60)	1.55 (1.30,2.20)	Z = 5.93	< 0.001
RR, insp/min, Mean ± SD	20.37 ± 6.40	20.09 ± 6.41	21.14 ± 6.34	t=-2.08	0.038
Height, cm, Mean ± SD	169.33 ± 14.96	169.36 ± 15.88	169.24 ± 12.13	t = 0.11	0.912
Weight, kg, M (Q_1_, Q_3_)	80.00 (68.00,95.30)	80.83 (69.35,95.60)	78.58 (66.45,94.60)	Z=-1.49	0.135
BMI, kg/m^2^, M (Q_1_, Q_3_)	27.61 (23.84,33.08)	27.82 (24.22,33.08)	26.77 (23.25,33.00)	Z=-1.83	0.067
Lymphocytes, %, M (Q_1_, Q_3_)	8.60 (5.00,13.20)	9.10 (5.30,14.00)	7.70 (4.00,11.00)	Z=-4.03	< 0.001
Neutrophil, %, Mean ± SD	79.02 ± 15.78	78.83 ± 14.95	79.55 ± 17.88	t=-0.54	0.592
Platelet, K/uL, M (Q_1_, Q_3_)	196.00 (128.00,278.00)	197.00 (135.00,283.00)	192.50 (115.00,269.00)	Z=-1.59	0.111
Glucose, mg/dL, M (Q_1_, Q_3_)	132.00 (107.00,174.00)	132.00 (107.00,171.00)	132.50 (107.00,181.00)	Z = 0.48	0.633
Follow-up time, day, M (Q_1_, Q_3_)	30.00 (23.00,30.00)	30.00 (30.00,30.00)	6.00 (2.00,13.00)	Z=-28.36	< 0.001
PNI, n (%)				χ^2^ = 22.86	< 0.001
PNI < 28.5	192 (23.10)	115 (18.88)	77 (34.68)		
PNI ≥ 28.5	639 (76.90)	494 (81.12)	145 (65.32)		
GNRI, n (%)				χ^2^ = 28.56	< 0.001
GNRI < 83.25	353 (42.48)	225 (36.95)	128 (57.66)		
GNRI ≥ 83.25	478 (57.52)	384 (63.05)	94 (42.34)		

AKI: acute kidney injury, ICU: intensive care unit, CCI: Charlson comorbidity index, SOFA: sequential organ failure assessment, SAPS-II: simplified acute physiology score II, GCS: glasgow coma scale, HB: hemoglobin, RDW: red blood cell distribution width, AG: anion gap, eGFR: esti mated glomerularfiltrationrate, SpO_2_: oxygen saturation, NLR: neutrophil lymphocyte ratio, PT: prothrombin time, INR: international normalized ratio, RR: respiratory rate, BMI: body mass index, PNI: prognostic nutritional index, GNRI: geriatric nutritional risk index

t: t test, χ^2^: chi-square test, Z: Whitney U rank sum test

### Correlation between PNI and GNRI and severity of AKI

We used eGFR, AKI stage, SOFA score and SAPS-II score to reflect the severity of AKI, and Table 2 showed the correlation between PNI and GNRI and the severity of AKI. The results showed that AKI stage (*r*_*s*_=-0.198), SOFA score (*r*_*s*_=-0.310) and SAPS-II score (*r*_*s*_=-0.276) were all negatively associated with PNI, while eGFR (*r*_*s*_=0.076) had a positive relationship. AKI stage (*r*_*s*_=-0.200), SOFA score (*r*_*s*_=-0.320) and SAPS-II score (*r*_*s*_=-0.300) were also negatively associated with GNRI.

**Table 2:** Correlation of PNI and GNRI and severity of AKI

Variables	eGFR		AKI stage		SOFA		SAPS-II	
	*r*_*s*_	*P*	*r*_*s*_	*P*	*r*_*s*_	*P*	*r*_*s*_	*P*
PNI	0.076	0.028	-0.198	< 0.001	-0.310	< 0.001	-0.276	< 0.001
GNPI	0.063	0.069	-0.200	< 0.001	-0.320	< 0.001	-0.300	< 0.001

PNI: prognostic nutritional index, GNRI: geriatric nutritional risk index, AKI: acute kidney injury, eGFR: esti mated glomerularfiltrationrate, SOFA: sequential organ failure assessment, SAPS-II: simplified acute physiology score II.

### Relationships between PNI and GNRI and 30-day mortality

Table S2 showed the covariates associated with 30-day mortality. After adjusting for the covariates, comparing to low PNI and GNRI levels, AKI patients who had high PNI [HR = 0.71, 95%CI: (0.51–0.698)] and GNRI [HR = 0.63, 95%CI: (0.47–0.86)] levels were seemed to have lower risk of 30-day mortality (Table 3).

**Table 3:** Association between PNI and GNRI and 30-day mortality and in age, SOFA, and SAPS-II subgroups

Subgroups	Variables	Model 1		Model 2		Model 3	
		h (95% CI)	*P*	HR (95% CI)	*P*	HR (95% CI)	*P*
	PNI^1^	0.51 (0.39–0.68)	< 0.001	0.48 (0.37–0.64)	< 0.001	0.71 (0.51–0.98)	0.036
	GNRI^2^	0.49 (0.38–0.64)	< 0.001	0.47 (0.36–0.61)	< 0.001	0.63 (0.47–0.86)	0.003
Age < 65	PNI^1^	0.54 (0.35–0.83)	0.005	0.49 (0.31–0.75)	0.001	1.14 (0.67–1.96)	0.622
	GNRI^2^	0.47 (0.31–0.72)	< 0.001	0.43 (0.28–0.66)	< 0.001	1.07 (0.65–1.75)	0.793
Age ≥ 65	PNI^1^	0.50 (0.35–0.71)	< 0.001	0.48 (0.34–0.69)	< 0.001	0.54 (0.35–0.82)	0.004
	GNRI^2^	0.50 (0.35–0.70)	< 0.001	0.48 (0.34–0.68)	< 0.001	0.51 (0.34–0.76)	< 0.001
SOFA < 6	PNI^1^	0.38 (0.21–0.67)	< 0.001	0.36 (0.20–0.65)	< 0.001	0.38 (0.18–0.81)	0.012
	GNRI^2^	0.57 (0.34–0.96)	0.035	0.52 (0.31–0.89)	0.016	0.57 (0.28–1.14)	0.110
SOFA ≥ 6	PNI^1^	0.69 (0.50–0.94)	0.021	0.67 (0.49–0.92)	0.013	0.80 (0.55–1.15)	0.223
	GNRI^2^	0.57 (0.42–0.78)	< 0.001	0.56 (0.41–0.77)	< 0.001	0.65 (0.46–0.93)	0.018
SAPS-II < 43	PNI^1^	0.30 (0.17–0.53)	< 0.001	0.25 (0.14–0.45)	< 0.001	0.22 (0.11–0.46)	< 0.001
	GNRI^2^	0.62 (0.36–1.08)	0.090	0.52 (0.29–0.90)	0.021	0.62 (0.32–1.22)	0.169
SAPS-II ≥ 43	PNI^1^	0.79 (0.58–1.09)	0.148	0.81 (0.59–1.13)	0.212	0.89 (0.62–1.28)	0.521
	GNRI^2^	0.61 (0.45–0.84)	0.002	0.63 (0.46–0.87)	0.005	0.63 (0.44–0.88)	0.008

PNI: prognostic nutritional index, GNRI: geriatric nutritional risk index, SOFA: sequential organ failure assessment, SAPS-II: simplified acute physiology score II, HR: hazard ratio, CI: confidence interval

1: PNI ≥ 28.5

2: GNRI ≥ 83.25

Model 1 was the crude model;

Model 2 adjusted for age, gender, and race;

Model 3 adjusted for age, gender, and race mechanical ventilation use, vasopressors use, AKI stage, ICU length of stay, CCI, SOFA, SAPS-II, HB, RDW, AG, eGFR, SpO2, NLR, PT, GCS, INR, and RR.

Notes: the variables to classify subgroup were not adjusted in its subgroup

We then explored these associations in subgroups of age, SOFA score, and SAPS-II score (Table 3). Among patients aged ≥ 65 years old, these negative relationships were also significant (all *P* < 0.05). In patients with SOFA score < 6 [HR = 0.38, 95%CI: (0.18–0.81)] or SAPS-II score < 43 [HR = 0.22, 95%CI: (0.11–0.46)], high PNI was associated with low risk of 30-day mortality, while in those who with SOFA score ≥ 6 [HR = 0.65, 95%CI: (0.46–0.93)] or SAPS-II score ≥ 43 [HR = 0.63, 95%CI: (0.44–0.88)], high GNRI was associated with low risk of 30-day mortality.

### The predictive performances of PNI and GNRI on 30-day mortality

**Fig. 2:**
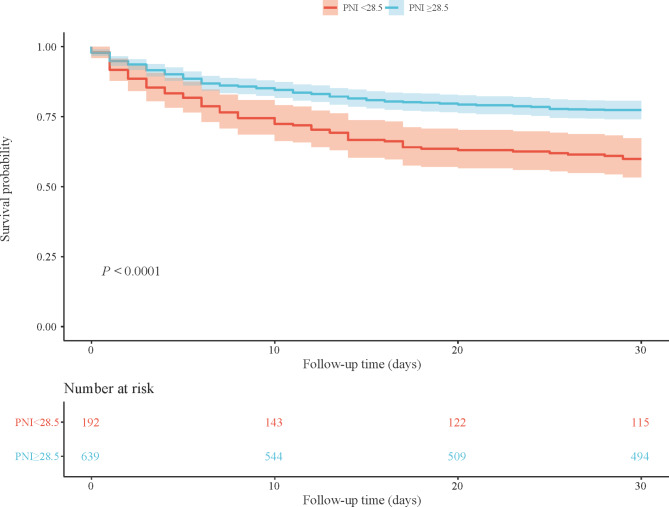
KM curve of the of the different PNI levels in AKI patients

**Fig. 3:**
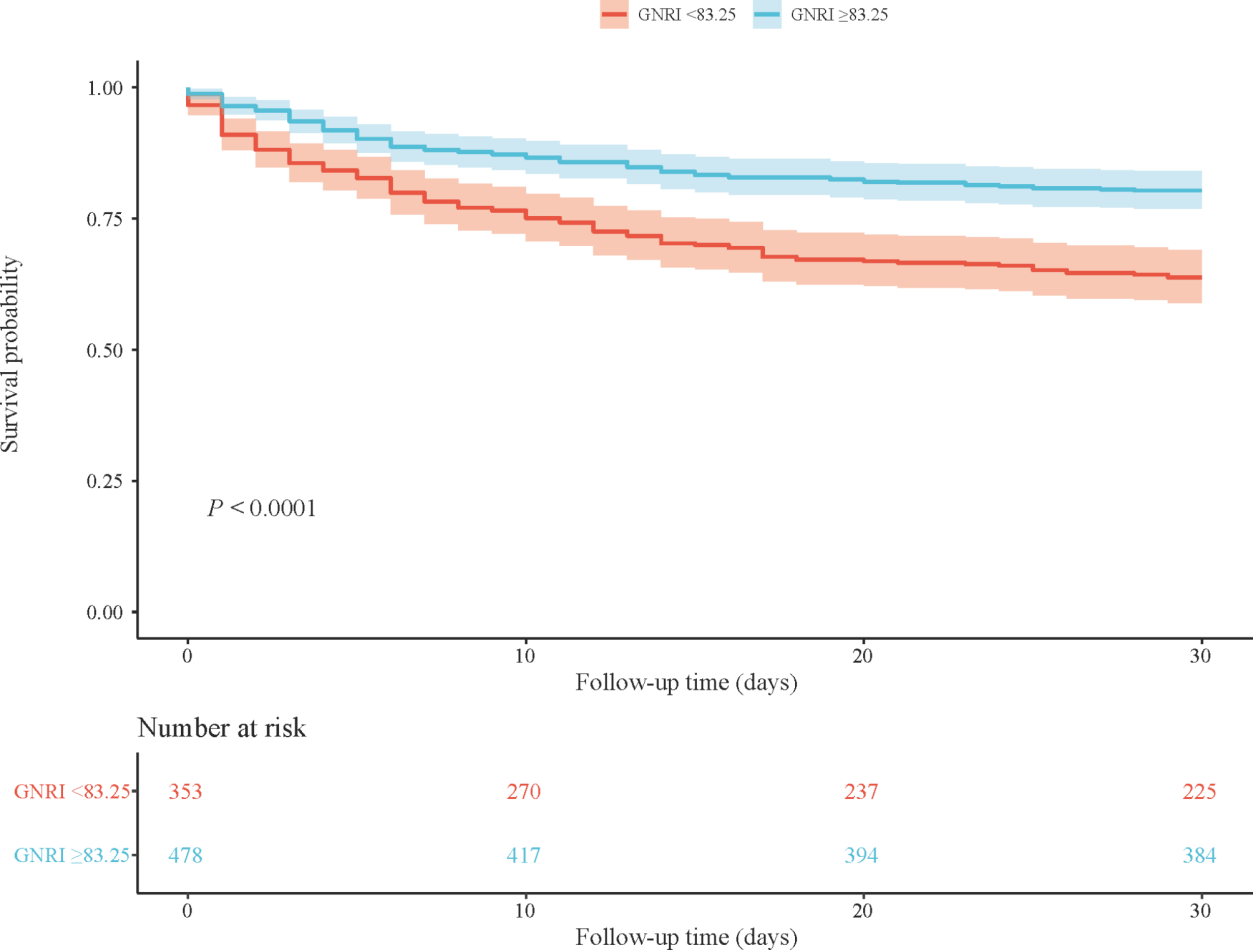
KM curve of the of the different GNRI levels in AKI patients

Figures 2 and 3 were respectively the KM curve of associations between PNI level and GNRI level and the survival probability in patients with AKI. The results showed that lower PNI and GNRI were both associated with a lower survival possibility (all *P* < 0.0001). Table 4 showed the C-index of PNI and GNRI on 30-day mortality, and the results indicated that PNI (C-index = 0.807) and GNRI (C-index = 0.806) may be potential predictors for 30-day mortality in patients with AKI.

**Table 4:** Predictive performance of PNI and GNRI on 30-day mortality in AKI patients

Variables	C-index
PNI	0.807
GNRI	0.806

PNI: prognostic nutritional index, GNRI: geriatric nutritional risk index, AKI: acute kidney injury

## Discussion

This study explored the relationships between PNI and GNRI and 30-day mortality in patients with AKI. Our results showed that high levels of PNI and GNRI were both associated with low risk of 30-day mortality. These relationships were also found in patients who aged ≥ 65 years old, with different SOFA scores and SAPS-II scores. Furthermore, it seemed that PNI and GNRI may be potential predictors for AKI in-hospital mortality.

To our knowledge, few studies have reported the predictive value of PNI for AKI prognosis. A pilot study by Shimoyama et al. [21] showed that PNI was a predictor of septic AKI prognosis. Hu et al. [22] suggested that the PNI may be a good predictor for identification of patients at high risk of AKI and mortality in the coronary care unit (CCU). PNI has been found as an independent risk factor for contrast-induced acute kidney injury (CI-AKI) [23], and was inversely and significantly associated with the development of CI-AKI in ST-elevation myocardial infarction (STEMI) [24]. Our research found that PNI ≥ 28.5 was significantly associated with low risk of 30-day mortality in patients with AKI, which may complement relevant research to a certain extent. Pathophysiological mechanisms of the relationship between high PNI and 30-day mortality in AKI have not been completely understood, and proinflammatory state may be a possible explanation. Inflammation was associated with increased catabolism and decreased albumin synthesis, and hypoalbuminemia may increase blood viscosity and disrupt endothelial function [25, 26]. Low lymphocyte count may be linked to increased inflammatory activity and pre-existing immunosuppression [27]. Therefore, the PNI basing on combination of serum albumin level and the lymphocyte count may be able to estimate the nutritional, immunity statuses and mortality of AKI patients in theory.

The predictive value of GNRI on AKI prognosis has also not been reported. Research by Seoudy et al. [28] indicated that low GNRI was related to an increased risk of all-cause mortality in patients received transcatheter aortic valve replacement (TAVR). A study on patients with acute respiratory distress syndrome (ARDS) showed that the GNRI on admission was linked to 30-day mortality, and may be a useful index to assess the mortality [29]. The current study has found that in AKI patients, high GNRI was associated with low risk of 30-day mortality. Serum albumin and BMI are two important components for the calculation of GNRI. Lacking of protein and amino acid supply in chronic malnutrition cases results in the formation of albumin reduction, and serum albumin concentration is reduced [28]. However, BMI has not been found to be associated with the mortality in patients with AKI in this research. Dietary sufficient/supplemented protein intake might influence serum albumin accompanied by weight gain, then the BMI is elevated, and finally the GNRI increases. Therefore, the mechanism of association between high GNRI and low risk of 30-mortality in AKI is needed further exploration.

In patients who aged ≥ 65 years old, high PNI and GNRI were both associated with low risk of 30-day mortality. In our study, patients who suffered from AKI were mostly with an old age (average aged 64.61 years old), in which ≥ 65 years old accounted for 51.87%. Previous studies demonstrated that malnutrition in older patients is common [30, 31]. The results of a research on comparing the predictive values of different nutritional risk assessment tools on perioperative clinical outcomes showed that the elderly patients undergoing elective spinal surgery, those who were diagnosed as malnutrition according to the PNI and GNRI, were at an increased risk for adverse events after surgery, and the GNRI had a better predictive power than the PNI [32]. Acarbaş et al. [33] found that older age and PNI < 47.7 were predictors of perioperative adverse events. In our opinion, GNRI and PNI may be potential predictors in AKI patients who aged ≥ 65 years old.

Mild AKI patients with SOFA score < 6 or SAPS-II score < 43 who had high PNI were seemed to have low risk of 30-day mortality. Differently, these negative association between GNRI and 30-day mortality were found in patients with severe AKI (SOFA score ≥ 6 and SAPS-II score ≥ 43). Malnutrition is prevalent in patients with AKI and is related to an increased in-hospital length of stay and all-cause mortality [34]. Critical illness is a hypermetabolic state, and consequently, patients with severe AKI are found to have an accelerated metabolic rate compared with patients with normal renal function [35]. The Society of Critical Care Medicine (SCCM) recommend using the Nutrition Risk in Critically III (NUTRIC) and Nutrition Risk Screening 2002 (NRS-2002) screening tools to assess nutrition risk in ICU patients [36]. The NUTRIC incorporates multiple variables, including age, number of comorbidities, SOFA scores, and number of days at hospital prior to admission in the ICU [36]. Patients with a higher NUTRIC scores are considered to be a strong positive association with higher 6-month mortality [37], but it is not frequently performed because of its high cost. Our results similarly found PNI and GNRI both had potential predictive values in AKI in-hospital mortality, but it may be affected by the age and severity assessment tools. Given that malnutrition has a strong association with in-hospital mortality, optimizing nutrition status is a key target for optimizing clinical care. How to select accurate nutritional status evaluation indicators in patients with different clinical conditions still needs further exploration.

This study explored the relationships between PNI and GNRI and 30-day mortality in patients with AKI, which may partly provide some references on potential predictors exploration in high-risk AKI patients. There are some limitations in the current research. The missing data were deleted, which may lead to overestimation of the outcome event. However, the results of sensitive analysis showed no significant difference of characteristics of participants before and after deletion of missing data. As a simple-center retrospective cohort study, selection bias is inevitable. It is difficult to adjust for all confounders such as the unknown intervention out of the ICU. Besides, the MIMIC-III database is short of other immunonutritional scores such as the Controlling Nutritional Status (CONUT) which could not be added to the predictive value comparison.

## Conclusion

PNI and GNRI may be potential predictors of 30-day mortality in patients with AKI. Further studies are needed to explore the exact association of PNI and GNRI with short-term mortality in patients with different conditions of AKI.

### Electronic supplementary material

Below is the link to the electronic supplementary material.

Supplementary Material 1

## Data Availability

The datasets generated and/or analyzed during the current study are available in the MIMIC-III database, https://mimic.physionet.org/iii/.

## Abbreviations

AKI: Acute kidney injury
PNI: prognostic nutritional index
GNRI: geriatric nutritional risk index
MIMIC-III: Medical Information Mart for Intensive Care III
ICU: intensive care unit
SpO_2_: oxygen saturation
NLR: neutrophil lymphocyte ratio
PT: prothrombin time
KDIGO: Kidney Disease Improving Global Outcomes
Scr: serum creatinine
CCI: Charlson comorbidity index
SOFA: Sequential Organ Failure Assessment
SAPS-II: Simplified Acute Physiology Score II
GCS: Glasgow Coma Scale
HB: hemoglobin
RDW: red blood cell distribution width
AG: anion gap
eGFR: esti mated glomerularfiltrationrate
INR: international normalized ratio
RR: respiratory rate
BMI: body mass index
HRs: hazard ratios
CIs: confidence intervals
KM: Kaplan-Meierwith
