# Supplementary material for: Association between nutritional status scores and the 30-day mortality in patients with acute kidney injury: an analysis of MIMIC-III database
Source: BMC Nephrol. 2023 Oct 6;24:296. doi: 10.1186/s12882-023-03329-5 (PMC10559585; doi:10.1186/s12882-023-03329-5)
Supplement: Supplementary file 1 — Supplementary Material 1 [file 12882_2023_3329_MOESM1_ESM.docx]

**Table S1. Sensitivity analysis of characteristics of patients before and after deletion of variables**

| Variables | Before deletion  (n=863) | After deletion  (n=831) | Statistics | *P* |
| --- | --- | --- | --- | --- |
| Age, years, Mean ± SD | 64.63 ± 15.93 | 64.61 ± 15.99 | t=0.02 | 0.981 |
| Gender, n (%) |  |  | χ^2^=0.03 | 0.862 |
| Female | 365 (42.29) | 348 (41.88) |  |  |
| Male | 498 (57.71) | 483 (58.12) |  |  |
| Race, n (%) |  |  | χ^2^=0.02 | 1.000 |
| Asian | 13 (1.51) | 12 (1.44) |  |  |
| White | 596 (69.06) | 576 (69.31) |  |  |
| Black | 56 (6.49) | 54 (6.50) |  |  |
| Hispanic | 22 (2.55) | 21 (2.53) |  |  |
| Others | 176 (20.39) | 168 (20.22) |  |  |
| Mechanical ventilation use, n (%) |  |  | χ^2^=0.08 | 0.776 |
| No | 286 (33.14) | 270 (32.49) |  |  |
| Yes | 577 (66.86) | 561 (67.51) |  |  |
| Vasopressors use, n (%) |  |  | χ^2^=0.00 | 0.963 |
| No | 433 (50.17) | 416 (50.06) |  |  |
| Yes | 430 (49.83) | 415 (49.94) |  |  |
| Renal replacement therapy, n (%) |  |  | χ^2^=0.02 | 0.894 |
| No | 839 (97.22) | 807 (97.11) |  |  |
| Yes | 24 (2.78) | 24 (2.89) |  |  |
| AKI stage, n (%) |  |  | χ^2^=0.16 | 0.922 |
| I | 153 (17.73) | 145 (17.45) |  |  |
| II | 390 (45.19) | 370 (44.52) |  |  |
| III | 320 (37.08) | 316 (38.03) |  |  |
| ICU length of stay, day, M (Q_1_, Q_3_) | 4.21 (2.49, 8.62) | 4.25 (2.50, 8.78) | Z=0.26 | 0.796 |
| CCI, M (Q_1_, Q_3_) | 2.00 (1.00, 4.00) | 2.00 (1.00, 4.00) | Z=0.15 | 0.883 |
| SOFA, M (Q_1_, Q_3_) | 6.00 (4.00, 9.00) | 6.00 (4.00, 9.00) | Z=0.50 | 0.620 |
| SAPS-II, M (Q_1_, Q_3_) | 43.00 (33.00, 54.00) | 43.00 (33.00, 55.00) | Z=0.37 | 0.712 |
| GCS, Mean±SD | 13.59 ± 2.93 | 13.57 ± 2.95 | t=0.12 | 0.902 |
| HB, g/dL, Mean±SD | 10.96 ± 2.20 | 10.95 ± 2.20 | t=0.05 | 0.961 |
| RDW, %, Mean ±SD | 15.46 ± 2.35 | 15.46 ± 2.32 | t=0.00 | 0.997 |
| AG, mEq/L, Mean±SD | 15.58 ± 4.74 | 15.60 ± 4.75 | t=-0.08 | 0.935 |
| eGFR, mL/min/1.73m^2^, M (Q_1_, Q_3_) | 67.83 (37.89, 97.54) | 66.81 (37.33, 96.98) | Z=-0.28 | 0.779 |
| SpO_2_, %, Mean±SD | 96.68 ± 4.33 | 96.65 ± 4.36 | t=0.14 | 0.885 |
| NLR, M (Q_1_, Q_3_) | 9.39 (5.80, 17.23) | 9.36 (5.80, 17.40) | Z=0.02 | 0.986 |
| PT, seconds, M (Q_1_, Q_3_) | 14.90 (13.50, 17.80) | 14.90 (13.50, 17.70) | Z=-0.03 | 0.977 |
| INR, M (Q_1_, Q_3_) | 1.40 (1.20, 1.70) | 1.30 (1.20, 1.70) | Z=-0.03 | 0.975 |
| RR, insp/min, Mean±SD | 20.37 ± 6.39 | 20.37 ± 6.40 | t=-0.01 | 0.989 |
| Height, cm, Mean±SD | 169.31 ± 14.82 | 169.33 ± 14.96 | t=-0.03 | 0.976 |
| Weight, kg, M (Q_1_, Q_3_) | 80.00 (68.00, 95.00) | 80.00 (68.00, 95.30) | Z=0.12 | 0.901 |
| BMI, kg/m^2^, M (Q_1_, Q_3_) | 27.53 (23.82, 33.08) | 27.61 (23.84, 33.08) | Z=0.11 | 0.914 |
| Lymphocytes, %, M (Q_1_, Q_3_) | 8.60 (5.00, 13.20) | 8.60 (5.00, 13.20) | Z=-0.08 | 0.935 |
| Neutrophil, %, Mean±SD | 79.02 ± 15.89 | 79.02 ± 15.78 | t=-0.00 | 0.996 |
| Platelet, K/uL, M (Q_1_, Q_3_) | 197.00 (130.00, 282.00) | 196.00 (128.00, 278.00) | Z=-0.25 | 0.801 |
| Glucose, mg/dL, M (Q_1_, Q_3_) | 132.00 (107.00, 173.00) | 132.00 (107.00, 174.00) | Z=0.16 | 0.873 |
| PNI, M (Q_1_, Q_3_) | 34.64 (29.04, 40.55) | 34.55 (28.90, 40.62) | Z=-0.19 | 0.851 |
| GNRI, Mean±SD | 84.71 ± 10.46 | 84.63 ± 10.42 | t=0.17 | 0.867 |
| 30-day mortality, n (%) |  |  | χ2=0.00 | 0.976 |
| No | 633 (73.35) | 609 (73.29) |  |  |
| Yes | 230 (26.65) | 222 (26.71) |  |  |
| Follow-up time, day, M (Q_1_, Q_3_) | 30.00 (23.00, 30.00) | 30.00 (23.00, 30.00) | Z=-0.04 | 0.965 |

AKI: acute kidney injury, ICU: intensive care unit, CCI: Charlson comorbidity index, SOFA: sequential organ failure assessment, SAPS-II: simplified acute physiology score II, GCS: glasgow coma scale, HB: hemoglobin, RDW: red blood cell distribution width, AG: anion gap, eGFR: esti mated glomerularfiltrationrate, SpO_2_: oxygen saturation, NLR: neutrophil lymphocyte ratio, PT: prothrombin time, INR: international normalized ratio, RR: respiratory rate, BMI: body mass index, PNI: prognostic nutritional index, GNRI: geriatric nutritional risk index.

t: t test, χ^2^: chi-square test, Z: Whitney U rank sum test.

**Table S2. Screening of covariates related to the 30-day mortality in AKI patients**

| Variables | HR (95% CI) | *P* |
| --- | --- | --- |
| Age | 1.01 (1.01-1.02) | 0.002 |
| Age group |  |  |
| Age <65 | Ref |  |
| Age ≥65 | 1.35 (1.04-1.77) | 0.027 |
| Gender |  |  |
| Female | Ref |  |
| Male | 1.25 (0.95-1.64) | 0.111 |
| Race |  |  |
| Asian | Ref |  |
| White | 1.64 (0.41-6.63) | 0.487 |
| Black | 1.34 (0.30-6.05) | 0.703 |
| Hispanic | 1.22 (0.22-6.64) | 0.822 |
| Others | 2.86 (0.70-11.67) | 0.143 |
| Mechanical ventilation use |  |  |
| No | Ref |  |
| Yes | 1.67 (1.22-2.27) | 0.001 |
| Vasopressors use |  |  |
| No | Ref |  |
| Yes | 2.52 (1.90-3.34) | <0.001 |
| Renal replacement therapy |  |  |
| No | Ref |  |
| Yes | 0.41 (0.13-1.29) | 0.127 |
| AKI stage |  |  |
| I | Ref |  |
| II | 1.29 (0.77-2.16) | 0.326 |
| III | 4.11 (2.54-6.63) | <0.001 |
| ICU length of stay | 0.97 (0.95-0.99) | 0.005 |
| CCI | 1.16 (1.10-1.23) | <0.001 |
| SOFA | 1.15 (1.12-1.19) | <0.001 |
| SAPS-II | 1.05 (1.04-1.05) | <0.001 |
| HB | 0.90 (0.85-0.96) | 0.001 |
| RDW | 1.16 (1.10-1.21) | <0.001 |
| AG | 1.06 (1.04-1.09) | <0.001 |
| eGFR | 0.99 (0.99-0.99) | <0.001 |
| SpO_2_ | 0.97 (0.95-0.99) | 0.018 |
| NLR | 1.01 (1.01-1.02) | <0.001 |
| PT | 1.01 (1.01-1.02) | <0.001 |
| GCS | 0.91 (0.88-0.95) | <0.001 |
| INR | 1.24 (1.16-1.33) | <0.001 |
| RR | 1.02 (1.01-1.04) | 0.043 |
| BMI | 0.99 (0.98-1.00) | 0.138 |
| Platelet | 1.00 (1.00-1.00) | 0.216 |
| Glucose | 1.00 (1.00-1.00) | 0.302 |
| PNI |  |  |
| PNI <28.5 | Ref |  |
| PNI ≥28.5 | 0.51 (0.39-0.68) | <0.001 |
| GNRI |  |  |
| GNRI <83.25 | Ref |  |
| GNRI ≥83.25 | 0.49 (0.38-0.64) | <0.001 |

AKI: acute kidney injury, HR: hazard ratio, CI: confidence interval, Ref: reference, ICU: intensive care unit, CCI: Charlson comorbidity index, SOFA: sequential organ failure assessment, SAPS-II: simplified acute physiology score II, HB: hemoglobin, RDW: red blood cell distribution width, AG: anion gap, eGFR: esti mated glomerularfiltrationrate, SpO_2_: oxygen saturation, NLR: neutrophil lymphocyte ratio, PT: prothrombin time, GCS: glasgow coma scale, INR: international normalized ratio, RR: respiratory rate, BMI: body mass index, PNI: prognostic nutritional index, GNRI: geriatric nutritional risk index.
